# Development and Initial Validation of a Measure of Body Image in the Postpartum Period: The Postpartum Body Image Scale

**DOI:** 10.3390/healthcare13222993

**Published:** 2025-11-20

**Authors:** Cristian Di Gesto, Marta Spinoni, Caterina Grano

**Affiliations:** Department of Psychology, Sapienza University of Rome, Via dei Marsi, 78, 00185 Rome, Italy; marta.spinoni@uniroma1.it (M.S.); caterina.grano@uniroma1.it (C.G.)

**Keywords:** postpartum body image scale, postpartum body image, postpartum body image concerns, postpartum positive body image, postpartum dieting

## Abstract

**Background**: Despite the importance of body image in the puerperium, tools specifically assessing postpartum body image are lacking. To address this gap, we developed the Postpartum Body Image Scale (PPBI-S), a measure encompassing both negative and positive key aspects of postpartum body image. **Methods**: The reliability and validity of the PPBI-S were evaluated across three studies. In Study 1, item formulation and content validity were established through expert evaluation and cognitive interviews (n = 20). Study 2 involved 250 postpartum women and employed exploratory factor analysis (EFA), revealing a trifactorial structure: Postpartum Body Image Concerns, Postpartum Positive Body Image, and Postpartum Dieting, accounting for 25.1%, 21.7%, and 17.1% of the variance, respectively. In Study 3, confirmatory factor analysis (CFA) was conducted on a sample of 258 postpartum women to examine internal consistency and convergent validity. **Results**: The CFA confirmed the three-factor model, showing good internal consistency across subscales. Convergent validity was demonstrated through significant correlations with established measures of body image and psychological well-being. Predictive validity was also supported, with PPBI-S scores significantly associated with breastfeeding intentions three months later. **Conclusions**: The PPBI-S is a reliable and valid instrument for assessing postpartum body image, capturing both its positive and negative dimensions. This tool provides researchers and clinicians with a comprehensive measure to evaluate body image experiences during the postpartum period.

## 1. Introduction

Body image is a multidimensional concept, that encompasses individuals’ perceptions, attitudes, feelings, and experiences related to their body size and shape [1,2], which can lead to altering one’s daily life [3,4,5]. During pregnancy, women undergo substantial changes [6,7] which may differently influence body image. Although weight gain and alterations in body shape, including those affecting the breasts, are natural changes in a healthy pregnancy, some women may experience negative reactions and stress to these adjustments which may impact quality of life, mental health, and well-being during pregnancy [8].

The literature evidenced a further intensification of body concerns during the postpartum period [8,9,10]. Indeed, research reported that postpartum women express more concerns about their bodies when compared to their counterparts in the pregnancy period [9,11] particularly due to increased worries about returning to pre-pregnancy size [12,13] which may be exacerbated by sociocultural pressure [14]. The concept of the bounce-back culture, which refers to the societal pressure on new mothers to rapidly regain their pre-pregnancy physique following childbirth, is characterized by unrealistic standards perpetuated by the media and prevailing social norms [15,16]. While changes in body shape and weight during pregnancy are generally perceived as normative by both the mother and society, given the expected weight gain associated with the fetus development [17,18], the transition to postpartum brings a shift in perspective, and body concerns may emerge [12,19,20].

This “bounce-back culture” thus epitomizes a sociocultural model that emphasizes appearance-based ideals and external evaluation, often neglecting the broader psychological and functional meaning of the maternal body. In recent years, however, scholars have advocated for a paradigm shift from a sociocultural to a psychological-functional perspective—one that recognizes the body not only for its appearance but also for its capabilities, resilience, and nurturing functions during the postpartum period [21].

Body image dissatisfaction during the postpartum period has been linked to adverse outcomes for both mothers and infants, including disordered eating patterns, namely excessive food restriction and restricted dieting [22] which pose nutritional risks also for the newborn development and may impair milk production and lead to energy deficiency [23,24,25].

Body dissatisfaction can influence also feeding practices shaping a woman’s choice to breastfeed due to concerns associated with the shape and size of the breast [26]. Indeed, maternal body image has been linked to decreased breastfeeding initiation and duration [27,28] and to an augmented use of formula feeding [28,29]. This is relevant as low breastfeeding rates are associated with an increased risk for childhood obesity that may extend into adulthood [30,31], and with an augmented risks for infections of the respiratory and gastrointestinal systems [32] along with significant health risks also for the mother including ovarian cancer [33], diabetes [34], and heart disease [35].

It is essential to recognize that body image not only involves individuals’ thoughts, feelings, and concerns regarding one’s physical appearance but, in a more positive view, also extends to perceptions of appreciation and functionality of the body [36]. In the realm of positive body image, body appreciation and functionality appreciation are interconnected facets [37,38]. Body appreciation involves harboring positive emotions, acceptance, and respect toward one’s own body, encompassing the acknowledgment and embrace of its unique physical attributes, strengths, and beauty [39,40]. The evaluation of body functionality encompasses all potential activities and capabilities of the body, aspects that have received limited attention in postpartum research [36]. This is noteworthy, as the postpartum period is marked by women directing their attention toward their bodies’ capabilities in safeguarding, nourishing, and facilitating the growth of their children [41]. Indeed, the appreciation of the body and its functionality, during the postpartum period, has been associated with the ability to start and maintain breastfeeding [42], and with other positive outcomes, including a reduction in postpartum depressive symptoms [41].

Overall, given the unique nature of concerns related to body dissatisfaction [43,44], the importance of eating practices during the postpartum period, along with the possible impact of newly appreciated body functions (such as breastfeeding), our objective was to develop a measure explicitly designed for assessing body image during this stage of life.

Despite the significant levels of reported dissatisfaction with body image in the postpartum period, the majority of studies examining maternal body image has primarily focused on the pregnancy phase. Moreover, existing questionnaires predominantly address general body concerns [e.g., Body Image Disturbance Questionnaire [45]; Body Shape Questionnaire [46]; Body Esteem Questionnaire [47], rather than specifically targeting body image disturbances related to peripartum. Only a prior investigation examined concerns during pregnancy regarding postpartum body image [29]. Nevertheless, the methodology employed in this study involved women who were asked to prospectively anticipate postpartum concerns without an actual assessment of these concerns during the postpartum. Hence, there remains a noticeable absence of tools specifically designed to assess postpartum body image.

A validated and specific instrument can help healthcare professionals and researchers identify early signs of body image dissatisfaction and related risk factors, contributing to the prevention of disordered eating behaviors and mood disturbances during the postpartum period. Furthermore, it may guide the design of targeted interventions aimed at promoting maternal mental health, fostering body appreciation, and supporting healthy adjustment to the postpartum body.

Recognizing this research gap, our study outlined the development and validation process of a self-report scale tailored to assess postpartum body image. In particular, based on Brown’s questionnaire on body image during pregnancy, we developed items to evaluate the dimensions both of body concerns and dieting, specially targeting postpartum women. Moreover, informed by the prior research conducted by Alleva et al. [48], we formulated items aimed at gauging positive body image during the postpartum phase. Measures of positive body image are fundamental, as psychologists need to extend beyond a pathology driven model and use these in their exploration of human strengths [49,50].

This work comprises three studies. In the first study (Study 1) we formulated the scale items and ascertained their content validity through evaluation by expert raters. Furthermore, we conducted cognitive interviews to evaluate the comprehension of the items involving a small sample of postpartum women. In the second study (Study 2) the structure of the scale was investigated through Explorative Factorial Analyses (EFA) to assess whether the items were loading onto the hypothesized factors. Finally, in the third study (Study 3), we performed a Confirmatory Factor Analysis (CFA) to examine the fit of the factorial structure identified in Study 2 in a further sample of postpartum women. Internal consistency and both convergent and predictive validity were also evaluated.

## 2. Study 1: Scale Development and Content Validity

The development of the items involved a multi-step process based on previous work about the development of scales related to body image, [51]: (1) defining the construct of postpartum body image and identifying its theoretical dimensions, considering both negative (i.e., body concerns) and positive body image constructs (i.e., body appreciation and functionality); (2) creating items based on the defined constructs of body image in the context of postpartum; (3) subjecting the items to analysis by expert raters; (4) selecting the most suitable items to represent the construct; (5) conducting a pre-test with the target population to assess cognitive understanding and clarity of the items. The 18 formulated items are displayed in Table 1.

Following the elaboration of the scale items, we sought evaluations from experts to assess the appropriateness of each item in relation to the dimensions for which they were designed (i.e., Postpartum Body Image Concerns, Postpartum Positive Body Image, and Postpartum Dieting).

## 3. Study 1a: Expert Analysis

### 3.1. Material and Methods

#### 3.1.1. Participants

The selection of experts was based on their expertise in studies related to body image. Contacts were established through email, wherein the study’s objectives were elucidated, and their cooperation was sought for the analysis of the items. Five experienced professional psychologists actively participated in this collaborative effort (Mage = 28.00; SD = 2.19).

#### 3.1.2. Measures

To assess the alignment between the items and the intended dimensions of postpartum body image, raters evaluated the 18 items based on specific criteria, as outlined in previous research [52,53]. The criteria included determining whether the item effectively represented its theoretical domain, accurately reflected that domain, presented its content clearly, and were relevant to the proposed dimension. Ratings were conducted on a 10-point scale, with 0 indicating a lack of appropriateness and 10 signifying complete appropriateness of the item to the aspects under consideration.

#### 3.1.3. Procedure

Participants were provided with access to an online survey designed to evaluate the specified criteria. The calculation of the Kappa coefficient was employed to assess the content appropriateness of the items and enhance the accuracy of item selection [54,55]. The Kappa coefficient serves to estimate the suitability of items across each dimension of the Postpartum Body Image Scale (PPBI-S), indicating the level of consensus among raters; a higher value signifies greater agreement. To gauge the accuracy, clarity, and relevance of the items, the Content Validity Coefficient (CVC) was computed. This coefficient gauges the consistency of each item among experts in terms of item content, with a higher coefficient suggesting increased uniformity among experts [53].

#### 3.1.4. Analytic Strategy

All analyses were conducted using the statistical software Jamovi, version 2.4.7 [56].

### 3.2. Results

Findings revealed a high level of agreement among raters, as indicated by an intraclass correlation coefficient (ICC) of 0.88, and a Kappa coefficient of 0.88 for item adequacy. In terms of accuracy, clarity, and relevance, the CVC were notably high: CVC Accuracy = 0.82; CVC Clarity = 0.84; CVC Relevance = 0.83. These findings provide preliminary evidence supporting the content validity of the PPBI-S items. Both the reliability of the items and the inter-rater agreement were reported, in accordance with methodologies outlined by Swami and Barron [57], suggesting that the items exhibit satisfactory quality for testing within the postpartum women population [58]. The expert raters did not propose any modifications to the formulation or wording of the items.

## 4. Study 1b: Cognitive Interview

### 4.1. Material and Methods

#### 4.1.1. Participants

Twenty-two postpartum women, who had given birth within the last three months, willingly volunteered to evaluate the clarity and understandability of the item wording. The age of the participating mothers ranged from 23 to 34 years (M = 27.18; SD = 1.12).

#### 4.1.2. Measures

The PPBI-S items were subjected to an analysis of content comprehension. During this phase, participants were asked to assess the clarity and understandability of the wording of each item, providing ratings on a scale from 1 (not clear) to 4 (completely clear).

#### 4.1.3. Procedure

Participants were contacted via email and briefed on the study’s objectives. Mean agreement scores for the comprehensibility of each item were calculated. The midpoint of the response scale (i.e., M > 2.5) served as the cutoff criterion for determining the appropriateness of the ratings.

### 4.2. Results

Mean scores for responses to each item fell within the range of 3.81 (SD = 1.09) to 4.01 (SD = 0.86), all of which were significantly higher than the midpoint of the response scale (i.e., M > 2.5; ts > 1.81 and < 9.22, at *p* < 0.001). These findings suggest that the items are semantically clear and are also well-understood by the target population.

### 4.3. Brief Discussion

This study aimed to develop the items for PPBI-S and to provide evidence of their content validity. The findings showed that participants perceived the items as appropriate, accurate, clear, and pertinent for gauging the three pivotal dimensions of postpartum women’s body image (i.e., Postpartum Body Image Concerns, Postpartum Positive Body Image, Postpartum Dieting). The involvement of expert raters in the content validity analysis played a pivotal role, aligning with established methodologies [53], ensuring the theoretical appropriateness of the items for their intended dimensions.

Furthermore, a critical step in this validation process involved a cognitive interview analysis to ascertain the comprehensibility of the item wording, ensuring clarity for the target population. Notably, no items were deemed necessary for exclusion, as neither experts nor participants proposed alterations to the presented content. This underscores the robustness and acceptance of the PPBI-S items in capturing the different dimensions of postpartum body image.

## 5. Study 2: Factorial Validity of the PPBI-S

The aim of this study was to examine whether the factorial structure of the PPBI-S aligns empirically with the three dimensions proposed for postpartum body image: Postpartum Body Image Concerns, Postpartum Positive Body Image, and Postpartum Dieting. EFA was employed to investigate the factorial structure of the scale.

### 5.1. Material and Methods

#### 5.1.1. Participants

A total of 508 mothers, who had given birth 1 to 3 months before, participated in the present study. Participants were randomly assigned to two subsamples using a random split procedure, the first, for Study 2, composed of 250 women (mean age = 30.18; SD = 3.41). In order to qualify for inclusion, they were required to be at least 18 years old and possess fluent Italian language skills. Sociodemographic details and childbirth information are provided in Table 2.

#### 5.1.2. Measures

Postpartum Body Image Scale. The development of the PPBI-S was based on a rational. We formulated items to capture facets of both negative and positive postpartum body image, drawing from insights identified in numerous theoretical contributions [42,59,60,61] about negative and positive body image among postpartum women. Specifically, the scale aimed to measure postpartum body image in the following domains: (i) body image concerns about the sizes and shapes of one’s body during postpartum, (7 items, e.g., “I worry that since I gave birth my body is less attractive than other women’s”), (ii) body appreciation and functionality (6 items, e.g., “I appreciate the different and unique characteristics of my body”), (iii) diet considerations for recovering the weight and physical shape prior to pregnancy (5 items, e.g., “It is important for me to get back to the weight I had before pregnancy”). The scale comprises 18 items, with responses on a 5-point Likert scale (1 = Strongly disagree; 5 = Strongly agree). Higher scores on the subscales of postpartum body image concerns, positive body image, and diet consideration correspond to greater levels of concern regarding one’s postpartum body image, higher appreciation of the body and of its functionality, and a heightened inclination toward dieting during the postpartum period, respectively. Completion of the PPBI-S requires approximately 3–5 min.

#### 5.1.3. Procedure

Participants were recruited through electronic mail, WhatsApp, Facebook, and other social platforms. Participants were invited to engage in an anonymous online survey administered via Qualtrics@ platform [62]. Participants provided informed consent, volunteered to participate and no incentives were offered. All procedures conducted in studies involving human participants adhered to the principles articulated in the 1964 Declaration of Helsinki and its subsequent amendments or equivalent ethical standards. To ensure that participants provided informed consent, they were provided with information about the study’s goals, data storage procedures, the voluntary nature of participation, and their ability to withdraw at any time. This information was all presented on the first page of the questionnaire. Participants retained the option to decline participation, and the study procedures received approval from the Institutional Review Board of the Department of Psychology to which the authors are affiliated.

#### 5.1.4. Analytic Strategy

##### Data Treatment

No responses were missing in the dataset as the questionnaire completion took place online, and responses to all questions were mandatory. A descriptive analysis of the scale items was carried out, considering statistical measures such as mean, standard deviation, skewness, and kurtosis. EFA was employed to assess the factorial structure, with the sample size for participants determined based on the number of items in the scale and the type of analyses conducted. Adhering to Nunnally’s recommendation [63], we considered a minimum of 10 cases per item. To ensure adequate sample sizes for EFA, a subset was randomly selected from the total sample, resulting in a sample of 250 participants for the EFA.

##### Exploratory Factor Analysis

The data underwent EFA using principal axis factorization with Varimax rotation. Prior to analysis, prerequisites such as the assessment of the correlation matrix [64] and the significance of Bartlett’s sphericity test [65] were considered. Principal axis factorization was selected due to its ability to provide results akin to the widely used maximum likelihood estimation, without assuming multivariate normality [66,67]. Factor loadings were deemed satisfactory if they exceeded 0.30 [68]. The determination of the number of extracted factors was based on eigenvalues exceeding 1.0 [69].

### 5.2. Results

#### 5.2.1. Item Analysis

Table 3 displays the descriptive statistics (mean, skewness, and kurtosis) for the 18 items of the PPBI-S.

All items of the PPBI-S exhibited skewness and kurtosis values below the recommended thresholds of 2.0 and 7.0, respectively, as outlined by Curran et al. [70]. Simulation studies have underscored significant methodological concerns when univariate skewness is ≥2.0 and kurtosis is ≥7.0 [70]. Based on the preliminary item analysis, all items were retained for the final factor extraction analysis.

#### 5.2.2. Exploratory Factor Analysis

Considering the item distribution, average correlation with other items, and item-total correlations [71], the data demonstrated suitability for factor analysis. The Kaiser-Meyer-Olkin (KMO) measure for sampling adequacy (0.95) and the significance of Bartlett’s test of sphericity (χ^2^_(153)_ = 4255, *p* < 0.001) in the present study indicated satisfactory factorability of the data. The EFA revealed a trifactorial structure of the scale, explaining 63.9% of the total variance. The subscales, Postpartum Body Image Concerns, Postpartum Positive Body Image, Postpartum Dieting, accounted for 25.1%, 21.7%, and 17.1% of the variance, respectively.

Factor loadings of the PPBI-S are presented in Table 4, with all items displaying values above 0.30 on the corresponding factor.

### 5.3. Brief Discussion

The present study provided preliminary evidence on the factorial structure of the PPBI-S. We retain eighteen items measuring three dimensions of postpartum body image that consider both negative and positive body image dimensions: postpartum body image concerns, postpartum positive body image, and postpartum dieting. Having a scale that measures these body image facets can allow for a better understanding of how these aspects play a role both in negative and positive postpartum body image. By identifying and measuring these dimensions, researchers can develop interventions, programs, or strategies to support women in improving their postpartum body image and overall well-being. Understanding the different aspects of postpartum body image can also help healthcare professionals provide more targeted and effective support to women during this important life stage.

## 6. Study 3: Confirmatory Analysis of the PPBI-S Factorial Structure

This study aims to extend the factorial validity analysis of the PPBI-S. We tested the hypothesis that the scale items reflect the multidimensional structure of PPBI-S as emerged from the EFA (i.e., Postpartum Body Image Concerns, Postpartum Positive Body Image, Postpartum Dieting).

Then, we estimated the convergent validity of the PPBI-S using measures that theoretically should be more strongly correlated with postpartum body image, namely the body shame subscale of the OBCS [72], the functionality appreciation scale [48], and the body shape questionnaire [73].

We also explored whether postpartum body image predicted breastfeeding mothers’ intentions measured after three months from the initial administration, providing evidence for the predictive validity of the PPBI-S.

### 6.1. Material and Methods

#### 6.1.1. Participants

The second subsample of 258 mothers (mean age = 29.19; SD = 2.17), drawn from a total sample of 508 mothers, who had given birth 1 to 3 months prior, participated in the current study. To meet the inclusion criteria, participants had to be at least 18 years old and proficient in the Italian language. Table 5 presents sociodemographic characteristics and childbirth-related data. A power analysis conducted using G*Power, version 3.1 [74] indicated that a minimum sample size of 130 would be required to detect small to medium effects (f = 0.30) with 80% power at an alpha level of 0.05.

#### 6.1.2. Measures

Postpartum Body Image. We used the 18 items of the PPBI-S developed in Studies 1 and 2 to measure postpartum body image.

Body Shame. The Body Shame subscale of the Italian version of the Objectified Body Consciousness Scale OBCS [72,75] was used. The subscale comprises 8 items (e.g., “I feel ashamed of myself when I haven’t made the effort to look my best”) with responses on a 7-point Likert scale (1 = Strongly disagree; 7 = Strongly agree) that measure body shame levels. Higher scores on this scale indicate greater levels of body shame. In the present study, the internal consistency of the scale was high (α = 0.92; ω = 0.91).

Functionality Appreciation. The Italian version of the Functionality Appreciation Scale FAS [48,76] was used. The scale comprises 7 items (e.g., “I appreciate my body for what it is capable of doing”) rated on a 5-point scale ranging from 1 = strongly disagree to 5 = strongly agree, which measures the levels of individual’s appreciation of one’s body for what it is capable of doing. Higher scores on this scale indicate greater levels of functionality appreciation. In the present study, the internal consistency of the scale was high (α = 0.91; ω = 0.92).

Body Dissatisfaction. The Italian version of the Body Shape Questionnaire-14 BSQ-14 [73,77] was used. The scale is composed of 14 items (e.g., “Have you felt that it is not fair that other people are thinner than you?”) rated on a 6-point Likert scale (1 = never; 6 = always). Participants were asked to respond referring to the last two weeks. Higher scores indicated higher levels of body dissatisfaction. In the present study, the internal consistency of the scale was high (α = 0.90; ω = 0.90).

Breastfeeding mother’s intentions. Three months after the initial administration (when infants were six months old) mothers’ intentions to continue breastfeeding in the first year of child life were assessed using a seven-item scale, adapted from previous research [78]. Respondents were instructed to indicate their level of agreement on a 7-point Likert scale (1 = not at all, 7 = very much).in response to statements related to the intention to breastfeed their child during the first year (e.g., “How likely is it that you intend to breastfeed your child till he/she is 12 months old?”). Higher scores on this scale indicated greater levels of mothers’ intentions to continue breastfeeding in the first year of child life (α = 0.89; ω = 0.90).

Body Mass Index (BMI). We calculated BMIs (kg/m^2^) using the participants’ reported weights and heights.

Sociodemographic and Obstetric Characteristics. Finally, socio-demographic information including age, education level, marital status, nationality, place of residence, and childbirth-related information including parity and type of delivery were collected.

#### 6.1.3. Analytic Strategy

##### Data Treatment

No responses were missing in the dataset as the questionnaire completion took place online, and responses to all questions were mandatory.

##### Preliminary Analyses

A preliminary item analysis was carried out to examine the descriptive statistics and the normality of the score’s distribution. Item distribution was first examined in order to identify items with excessive skewness (>2) and kurtosis (>7) values [79].

##### Confirmatory Factor Analysis

With a different subsample (n = 258), a CFA was performed. Models fit was assessed using the following indices: (i) relative/normed chi-square (χ^2^/df), with lower values indicating better model fit [80,81] [regarding the χ^2^ value, poor fit on a small sample might result in a non-significant χ^2^, while good fit based on a larger sample might result in a significant χ^2^; however, χ^2^/df minimizes the impact of sample size on χ^2^]; (ii) Comparative Fit Index (CFI); (iii) Tucker–Lewis Index (TLI); (iv) Root Mean Square Error of Approximation (RMSEA); (v) Standardized Root Mean Square Residual (SRMR). RMSEA and SRMR values of 0.05 and 0.08, respectively, were considered for good and moderate fit [80], and CFI and TLI values of 0.90 and 0.95, respectively, were considered for good and excellent fit [80]. Additionally, for CFA, factor loadings were deemed appropriate if they exceeded 0.30 [68].

##### Reliability and Validity Analysis

To evaluate internal consistency, Cronbach’s alphas (α) were computed for the PPBI-S subscales. Alpha values ranging from 0.70 to 0.90 are considered excellent, while those around 0.60 are deemed good [82].

Convergent validity was assessed through bivariate Pearson correlations (r) between the PPBI-S subscales (i.e., Postpartum Body Image Concerns, Postpartum Positive Body Image, Postpartum Dieting), and body shame (Body Shame subscale of the OBCS), functionality appreciation (FAS), and body dissatisfaction (BSQ-14). Relationships between variables were classified as small if around 0.10, moderate if around 0.30, and strong if 0.50 or above [83].

Predictive validity was investigated via a linear regression model, employing breastfeeding intentions measured after three months from the initial administration as the criterion variable, and the three dimensions of postpartum body image (i.e., Postpartum Body Image Concerns, Postpartum Positive Body Image, Postpartum Dieting) as potential predictors. A linear regression analysis using the enter method was performed to ascertain which factors predicted intentions to breastfeed in the ensuing 12 months.

### 6.2. Results

#### 6.2.1. Item Analysis

Descriptive statistics (mean, skewness, and kurtosis) for the 18 items of the PPBI-S are presented in Table 6.

All items of the PPBI-S showed skewness and kurtosis values below the recommended thresholds of 2.0 and 7.0, respectively [70]. All items were included in the final factor extraction analysis based on the preliminary item analysis.

#### 6.2.2. Confirmatory Factor Analyses

**Figure 1:**
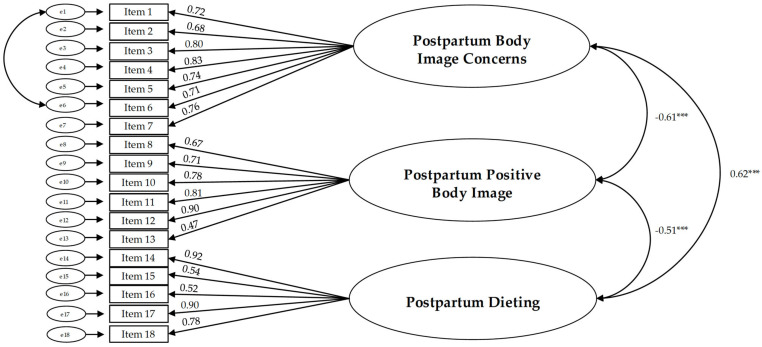
Confimatory factor analysis model, factor loadings and correlations between factors (n = 258). *** *p* < 0.001.

To validate the factor structure identified through the EFA, a CFA was conducted on a subsample consisting of 258 postpartum women, testing the three-factor model. The results indicated that the fit indices were not entirely acceptable (χ^2^/df = 4.65, *p* < 0.001; CFI = 0.87, TLI = 0.88, RMSEA [90% CI] = 0.071 [0.060; 0.101], SRMR = 0.062). Modification indices suggested adding a covariance between the errors of items 1 and 6, both of which belong to the Postpartum Body Image Concerns factor. Content analysis of each item revealed considerable overlap, attributed to the item wording effect [84,85], between item 1 (“I am concerned that since I gave birth, I feel less attractive”) and item 6 (“I am worried that since I gave birth, my body is less attractive compared to other women.”). This overlap in item wording can lead to spurious covariances [86]. The modified three-factor model exhibited a good fit (χ^2^/df = 5.71, *p* < 0.001; CFI = 0.94, TLI = 0.93, RMSEA [90% CI] = 0.081 [0.053; 0.081], SRMR = 0.041). Factor loadings for the three subscales are reported in Figure 1. Each item loaded strongly onto its intended factors. Standardized factor loadings ranged from 0.47 to 0.92, and the level of significance was below 0.001. Correlations between the three factors were: r = −0.61 (*p* < 0.001) between Postpartum Body Image Concerns and Postpartum Positive Body Image; r = 0.62 (*p* < 0.001) between Postpartum Body Image Concerns and Postpartum Dieting, and r = −0.51 (*p* = 0.001) between Postpartum Positive Body Image and Postpartum Dieting.

#### 6.2.3. Reliability

As for reliability, for the PPBI-S subscales (Postpartum Body Image Concerns: α = 0.92, Postpartum Positive Body Image: α = 0.90, Postpartum Dieting: α = 0.88), the levels of internal consistency are excellent.

#### 6.2.4. Convergent and Predictive Validity

Regarding convergent validity, from the analysis of bivariate correlations, it emerged that scores on the Postpartum Body Image Concerns subscale of the PPBI-S were strongly and positively associated with scores of the Body Shame Subscale of the OBCS, and the association was statistically significant (r = 0.66, *p* < 0.001). Additionally, a robust and statistically significant positive correlation was found between scores of the Postpartum Positive Body Image subscale of the PPBI-S and scores of the FAS subscale (r = 0.65, *p* < 0.001). Finally, the association between scores of the Postpartum Dieting subscale of the PPBI-S and scores of the BSQ-14 was strong, positive, and statistically significant (r = 0.52, *p* < 0.001).

Regarding predictive validity, the linear regression analysis indicated that all three subscales of the PPBI-S were predictive of breastfeeding intentions. The scores related to the subscales Postpartum Body Image Concerns (β = −0.21, *p* < 0.001) and Postpartum Dieting (β = −0.18, *p* < 0.001) significantly and negatively predicted participants’ intentions for breastfeeding (F_(2,248)_ = 7.12, *p* < 0.001). Postpartum Positive Body Image subscale predicted breastfeeding intentions significantly and positively (β = 0.19, *p* < 0.001).

The percentage of variance explained by the regression model was 26%.

### 6.3. Brief Discussion

The present study provided empirical support for the factorial, convergent, and predictive validity of the PPBI-S. Analysis of the findings confirmed a three-factor structure for the PPBI-S. The instrument was found to effectively assess distinct facets of postpartum body image. The PPBI-S demonstrated the capacity to capture women’s worries regarding postpartum body image, postpartum positive body image, and dietary behaviors, offering valuable insights for future research to differentiate specific domains more comprehensively. The items within each subscale exhibited satisfactory internal consistency for measuring the respective dimensions. Additionally, all three subscales showed internal consistency and good convergent validity with measures of negative body image (Body Shame Subscale of the OBCS and BSQ-14) and positive body image (FAS), as well as good predictive validity concerning intentions to breastfeed, measured after three months from the first administration.

## 7. General Discussion

Several scholars have highlighted the existence of a gap in the postpartum body image literature, indicating a lack of both theoretical and empirical foundations among professionals for comprehending negative and positive postpartum body image and associated variables [8,9]. To address this gap and contribute to the research, it is crucial to create and evaluate measures that concurrently encompass both negative and positive aspects of postpartum body image [27]. Therefore, we developed a measure of postpartum body image, namely the PPBI-S, incorporating key facets of both negative and positive body image, and examined its psychometric properties, reliability, and validity, across three studies.

In Study 1, we formulated the items of the PPBI-S and analyzed the scale’s content validity. In Study 2, we evaluated the factorial structure of the scale and the three hypothesized dimensions of postpartum body image (i.e., Postpartum Body Image Concerns, Postpartum Positive Body Image, Postpartum Dieting) emerged. In Study 3, to validate the factor structure identified through the EFA in Study 2, a CFA was conducted on a distinct subsample of postpartum women. Moreover, we investigated the convergent validity of the PPBI-S with well-established measures of body shame, functionality appreciation, and body concerns. Finally, when the infants were six months old, we evaluated the predictive validity of the scale in relation to mothers’ intentions to continue breastfeeding during the first year of the child’s life.

With regard to Study 1, we developed the items for PPBI-S and provided evidence of its content validity. The results showed that participants perceived the items as appropriate, accurate, clear, and pertinent for gauging the three pivotal dimensions of postpartum women’s body image. This contributes to the trustworthiness of the study’s results. A well-perceived measurement tool is crucial for obtaining meaningful and representative data, which is essential for informing interventions, clinical practices, and further research in the field of postpartum psychology. Moreover, the inclusion of expert raters in the content validity analysis, in accordance with established methodologies [53], ensured the theoretical appropriateness of the items for their intended dimensions. Furthermore, adopting a cognitive interview analysis in the validation process served to emphasize the importance of item wording comprehensibility. This step ensured clarity for the target population, contributing to the overall robustness of the PPBI-S. The fact that no items were deemed necessary for exclusion, and neither experts nor participants proposed alterations, highlights the high level of acceptance and validity of the PPBI-S items. This solidifies the instrument’s efficacy in accurately capturing the different dimensions of postpartum body image, further emphasizing its utility in advancing research and clinical understanding in this domain. This approach enhances the reliability of the measurement tool and underscores its utility in capturing the complexities of postpartum body image, that can arise from the multitude of factors that influence a woman’s perception of her body after giving birth [87,88]. This complexity stems from various physical, emotional, social, and psychological changes that occur during the postpartum period [9,89]. By taking a multidimensional approach to measuring postpartum body image, researchers may develop a comprehensive understanding of the complexities involved in how women view their bodies after childbirth, which in turn may better inform interventions and support strategies.

Regarding Study 2, from the exploratory analysis of the factorial structure, the hypothesized three-factor structure emerged. The Postpartum Body Image Concerns subscale accounts for 25.1% of the variance, the Postpartum Positive Body Image subscale for 21.7%, and the Postpartum Dieting for 17.1%. The three subscales showed excellent internal consistency (α range: 0.89–0.93).

The structure that emerged in Study 2 was confirmed in Study 3 through CFA, yielding fit indices within acceptable thresholds, thus supporting the adequacy of the three-factor model. The items within each subscale exhibited satisfactory internal consistency for measuring the respective dimensions emerged in Study 2. Moreover, this third study provided preliminary evidence on the convergent validity of the PPBI-S. Indeed, the subscales of the PPBI-S showed good concurrent validity with well-established measures of negative body image (i.e., body shame and body dissatisfaction) and positive body image (i.e., functionality appreciation). Notably, the Postpartum Body Image Concerns subscale of the PPBI-S was strongly and positively associated with scores of the Body Shame Subscale of the OBCS. This association can be attributed to the theoretical underpinnings of body image and shame constructs within the context of postpartum experiences. Postpartum body image concerns often revolve around changes in physical appearance and self-perception following childbirth [8,90]. These concerns are closely linked to societal norms and expectations regarding body image. The so-called bounce-back culture reflects the expectation that new mothers should promptly recover their pre-pregnancy bodies, a pressure reinforced by unrealistic beauty ideals and widespread media narratives [16]. The discrepancy between these societal expectations and the reality of the postpartum experience further complicates women’s perceptions of their bodies, highlighting the intricate interplay between external influences and internal struggles in shaping postpartum body image [16,42]. This emphasis on quickly bouncing back to one’s pre-pregnancy body, places significant psychological and emotional strain on new mothers, contributing to feelings of inadequacy and heightened unpleasant emotions related to one’s own body, like shame [29,91]. From a theoretical perspective, this dynamic can also be framed within the self-objectification theory [92], according to which sociocultural pressures lead women to internalize an observer’s perspective on their bodies, promoting self-monitoring and body shame. In the postpartum period, this internalized objectifying gaze may intensify, as women are exposed to societal ideals of rapid recovery and attractiveness, which reinforce appearance-based evaluation over bodily functionality.

Body shame is a complex emotional response characterized by feelings of inadequacy, self-criticism, and negative evaluation of one’s body [93]. Given the intimate connection between body image concerns and body shame, it is not surprising that individuals experiencing heightened postpartum body image concerns may also exhibit higher levels of body shame. The association between these constructs may also reflect the internalization of societal beauty standards, fear of judgments and comparison with idealized images [94,95].

The strong positive association found between the Postpartum Positive Body Image subscale of the PPBI-S with a well-validated scale that measures positive aspects of body image (i.e., FAS) confirms the convergent validity of this subscale. Finally, the positive correlation of the Postpartum Dieting subscale with the Body Shape Questionnaire which evaluates concerns about body shape that may lead to difunctional eating patterns, confirms the convergent validity also for this subscale. This is in line with studies that show a positive association between dietary behaviors and levels of body dissatisfaction in the postpartum period [12,96]. Such reciprocal association highlights the complex dynamic between postpartum dietary habits and body image concerns [97].

Study 3 also demonstrated the good predictive validity of the PPBI-S. Findings showed that Postpartum Body Image Concerns and Postpartum Dieting subscales scores significantly and negatively predicted mothers’ breastfeeding intentions in the first year of child’s life. This could be attributed to the societal pressures and expectations to return to pre-pregnancy body shape and size, which lead women to prioritize dieting practices and appearance over breastfeeding [16,29,42].

Conversely, Postpartum Positive Body Image subscale predicted breastfeeding intentions positively and significantly, suggesting that mothers who value and appreciate their body and its functional aspects may be more inclined to have positive intentions toward breastfeeding. This finding underscores the importance of considering not only body image concerns and dieting behaviors but also the broader perception of postpartum positive body image in understanding and promoting breastfeeding intentions among new mothers [42,60]. Further research exploring the relationship between postpartum positive body perceptions and breastfeeding experience is warranted to inform targeted interventions and support strategies for promoting successful breastfeeding outcomes.

Overall, the introduction of the PPBI-S stands as a novel advancement to assess and explore the complexities of postpartum body image, filling a gap in existing research where no comparable instrument has been developed before. Considering the centrality of body image in the postpartum period [12,20], this new scale provides a reliable and valid measure to specifically assess the various facets of body image in postpartum, including an underexplored construct concerning postpartum positive body image. Appreciation of the body and its functionality includes favorable opinions of the body, acceptance of imperfections, and respect for the novel needs and function of own body [51]. By appreciation of one’s body and by valuing its functional facets, women can experience a healthier peripartum, prevent potential complications, and enhance their overall mental well-being [60].

The strong psychometric properties of the scale, along with the findings of good convergent and predictive validity, are promising for its practical relevance in assessments, clinical interventions, and research settings. Indeed, early identification of negative and positive body image issues in postpartum women could lead to promptly referring them to appropriate interventions or support services.

### Limitations and Strengths

Although all the present study’s findings are consistent, some limitations must be considered. For example, first, the scale’s reliability was assessed solely in relation to internal consistency. Future longitudinal research designs could examine the temporal stability of PPBI-S scores by incorporating a test–retest evaluation. Second, the potential for bias was inherent in voluntary participation, possibly favoring women who were more inclined to report postpartum body image experiences. Future research may employ more representative sampling designs. Third, the oversight of social desirability levels represents a limitation; thus, forthcoming studies could address this by incorporating controls for this variable. Moreover, it should be noted that all data collected in the present validation studies were based on self-report measures and a cross-sectional design, which may limit the ability to establish temporal or causal relationships among variables. Future longitudinal studies could therefore provide a more comprehensive understanding of the stability and predictive power of the PPBI-S over time. Additionally, future studies should examine the measurement invariance of the PPBI-S across more diverse samples (e.g., age, socioeconomic status, nationality) to ensure that the instrument performs equivalently and maintains its validity across different groups of postpartum women. Given that the present validation was conducted with an Italian sample, further research is needed to confirm the generalizability of the PPBI-S across different cultural contexts. Cross-cultural validation would allow for the assessment of potential cultural variations in postpartum body image experiences and ensure the scale’s applicability in international research and clinical practice.

Despite these limitations, the three studies conducted led to the development of a valid and reliable instrument that considers both negative and positive postpartum body image dimensions. The developed measure holds promise as a valuable tool for clinicians and researchers alike, providing a nuanced assessment of postpartum body image that can inform intervention strategies and contribute to the holistic well-being of postpartum women.

This study has significant strengths. Firstly, our results provide new evidence confirming the validity and reliability of an instrument aimed at measuring postpartum body image among mothers of newborns in the Italian context. Results suggest good convergent, divergent and predictive validity for the PPBI-S. Furthermore, the present study demonstrates that the scale exhibits excellent internal consistency. This is particularly positive, considering the wide range of possible research and clinical applications for such a tool in the postpartum body image context [14,20]. Specifically, the PPBI-S can be employed in clinical settings as an early screening tool for identifying women at risk of developing body image disturbances or related psychological difficulties, and to evaluate the effectiveness of targeted interventions promoting maternal mental health and body appreciation. In addition, the scale’s robust psychometric properties and theoretical grounding make it a promising instrument for cross-cultural research, allowing comparisons of postpartum body image experiences across different sociocultural contexts.

Finally, given its reasonable brevity and adaptability, the PPBI-S could be useful for researchers and healthcare professionals in contexts requiring a simple, quick, and efficient assessment of postpartum body image issues.

## 8. Conclusions

The PPBI-S represents the first specific measure for postpartum body image. The results highlight good psychometric properties. Notably, aside from its excellent internal consistency, the PPBI-subscales exhibit good convergent validity. Furthermore, while the Postpartum Body Image Concerns and Postpartum Dieting subscales predict decreased intentions for breastfeeding in the first year of the child’s life, the Postpartum Positive Body Image Scale positively predicts such intentions. These findings underscore the utility of the scale in identifying risk and protective factors towards optimal breastfeeding intentions and inform targeted interventions to support and promote breastfeeding practices. The relative brevity of the PPBI-S makes it agile and suitable for various contexts, especially clinical settings, allowing for an early assessment of body image symptoms, facilitating secondary prevention interventions, and guiding clinical interventions to support women facing postpartum body image difficulties. Notably, the PPBI-S may be integrated into postpartum assessments as a screening tool to identify women experiencing heightened body image concerns, maladaptive dieting behaviors, or low appreciation of their body functionality. Its use may support clinicians in tailoring interventions aimed at reducing body dissatisfaction and promoting more positive and functional body perceptions. Moreover, the PPBI-S could serve as an outcome measure to evaluate the efficacy of programs designed to enhance women’s adjustment to postpartum changes, support breastfeeding maintenance, and foster psychological well-being. In particular, the PPBI-S could be effectively implemented within multidisciplinary postpartum support programs—including psychological counseling, nutritional guidance, and lactation support—to monitor progress, identify specific risk factors, and promote integrated care approaches addressing both physical and emotional aspects of maternal well-being.

## Figures and Tables

**Table 1:** Items of the Postpartum Body Image Scale—PPBI-S.

Item	English Version	Italian Version
1	Since giving birth, I feel less attractive.	Mi preoccupo del fatto che da quando ho partorito mi sento meno attraente.
2	I worry that my partner finds me less attractive since I gave birth.	Mi preoccupo che il mio partner mi trovi meno attraente da quando ho partorito.
3	I worry that my postpartum breasts are less attractive.	Mi preoccupo che il mio seno dopo il parto sia meno attraente.
4	I worry that my postpartum belly is less attractive.	Mi preoccupo che la mia pancia dopo il parto sia meno attraente.
5	I worry that my postpartum body is less attractive compared to before pregnancy.	Mi preoccupo che il mio corpo dopo il parto sia meno attraente rispetto a prima della gravidanza.
6	I worry that since giving birth my body is less attractive compared to other women’s bodies.	Mi preoccupo che da quando ho partorito il mio corpo sia meno attraente rispetto a quello di altre donne.
7	I worry that other people find my postpartum body less attractive.	Mi preoccupo che altre persone trovino il mio corpo dopo il parto meno attraente.
8	I appreciate what my postpartum body is allowing me to do.	Apprezzo quello che il mio corpo dopo il parto mi sta permettendo di fare.
9	I feel good in my postpartum body at this moment.	Mi sento bene nel mio corpo dopo il parto in questo momento.
10	I look at my postpartum body with a positive attitude.	Guardo con un atteggiamento positivo al mio corpo dopo il parto.
11	I am attentive to the nutritional needs of my postpartum body.	Sono attenta ai bisogni nutrizionali del mio corpo dopo il parto.
12	My postpartum body makes me feel good.	Il mio corpo dopo il parto mi fa sentire bene.
13	I am happy with my postpartum body.	Sono contenta del mio corpo dopo il parto.
14	I should go on a diet to lose weight.	Dovrei fare una dieta per perdere peso.
15	It would be necessary for me to exercise to lose weight.	Sarebbe necessario che io facessi attività fisica per perdere peso.
16	It is necessary for me to regain my physical fitness and ideal weight.	È necessario per me recuperare la mia forma fisica e il mio peso ideale.
17	It is important for me to return to the weight I had before pregnancy.	È importante per me tornare al peso che avevo prima della gravidanza.
18	It would be appropriate for me to do something to return to my pre-pregnancy physical fitness.	Sarebbe opportuno che io facessi qualcosa per tornare alla mia forma fisica precedente la gravidanza.

Note: Study 1. Hypothesized dimensions of postpartum body image: Postpartum Body Image Concerns (items 1–7), Postpartum Positive Body Image (items 8–13), and Postpartum Dieting (items 14–18).

**Table 2:** Sociodemographic and birth-related characteristics (n = 250).

Variables		Mean ± SD or %
Age		30.18 ± 3.41
Education level		
	Middle school	12.3%
	High school	28.9%
	Bachelor degree	23.4%
	Master’s degree	18.7%
	Post lauream specialization courses	9.6%
	Ph.D.	7.1%
Marital status		
	Unmarried	2.4%
	Married/cohabiting	85.7%
	Separated/divorced	11.9%
Nationality		
	Italian	98%
	Foreign	2%
Place of residence		
	Italy	100%
Parity		
	Primiparous	76.4%
	Multiparous	23.6%
Type of delivery		
	Spontaneous vaginal delivery	58.2%
	Induced vaginal delivery	19.1%
	Elective cesarean section	18.5%
	Emergency cesarean section	4.2%

Note: Study 2.

**Table 3:** Descriptive statistics for the 18 items of PPBI-S (n = 250).

Item	Mean (SD)	Asymmetry (SD)	Kurtosis (SD)
1	3.12 (1.43)	−0.11 (0.14)	−1.25 (0.28)
2	2.96 (1.46)	−0.03 (0.14)	−1.36 (0.28)
3	2.79 (1.47)	0.20 (0.14)	−1.29 (0.28)
4	3.55 (1.42)	−0.51 (0.14)	−1.03 (0.28)
5	3.23 (1.41)	−0.18 (0.14)	−1.20 (0.28)
6	2.89 (1.50)	0.11 (0.14)	−1.35 (0.28)
7	2.52 (1.43)	0.44 (0.14)	−1.02 (0.28)
8	3.85 (1.17)	−0.76 (0.14)	−0.15 (0.28)
9	2.91 (1.25)	0.04 (0.14)	−0.90 (0.28)
10	3.07 (1.27)	−0.05 (0.14)	−0.84 (0.28)
11	3.13 (1.17)	−0.10 (0.14)	−0.64 (0.28)
12	2.79 (1.09)	0.01 (0.14)	−0.50 (0.28)
13	2.67 (1.21)	0.12 (0.14)	−0.75 (0.28)
14	3.66 (1.44)	−0.72 (0.14)	0.86 (0.28)
15	3.85 (1.36)	−0.94 (0.14)	−0.44 (0.28)
16	3.87 (1.26)	−0.89 (0.14)	−0.26 (0.28)
17	3.62 (1.41)	−0.64 (0.14)	−0.89 (0.28)
18	3.76 (1.32)	−0.79 (0.14)	−0.52 (0.28)

Note: Study 2.

**Table 4:** Factor loadings of the 18 items of PPBI-S (n = 250).

	Factors		
Item	Postpartum Body Image Concerns	Postpartum Positive Body Image	Postpartum Dieting
1	0.76		
2	0.74		
3	0.51		
4	0.74		
5	0.78		
6	0.71		
7	0.63		
8		0.56	
9		0.68	
10		0.82	
11		0.60	
12		0.78	
13		0.79	
14			0.69
15			0.78
16			0.67
17			0.65
18			0.69

Note: Study 2.

**Table 5:** Sociodemographic and birth-related characteristics (n = 258).

Variables		Mean ± SD or %
Age		30.18 ± 3.41
Education level		
	Middle school	12.1%
	High school	28.8%
	Bachelor’s degree	23.4%
	Master’s degree	18.6%
	Post lauream specialization courses	9.4%
	Ph.D.	7.7%
Marital status		
	Unmarried	2.1%
	Married/cohabiting	85.9%
	Separated/divorced	12%
Nationality		
	Italian	96.8%
	Foreign	3.2%
Place of residence		
	Italy	100%
Parity		
	Primiparous	80.7%
	Multiparous	19.3%
Type of delivery		
	Spontaneous vaginal delivery	60.1%
	Induced vaginal delivery	18.3%
	Elective cesarean section	18%
	Emergency cesarean section	3.6%

Note: Study 3.

**Table 6:** Descriptive statistics for the 18 items of PPBI-S (n = 258).

Item	Mean (SD)	Asymmetry (SD)	Kurtosis (SD)
1	3.10 (1.39)	−0.12 (0.17)	−1.22 (0.34)
2	2.91 (1.43)	−0.02 (0.17)	−1.37 (0.34)
3	2.78 (1.42)	0.21 (0.17)	−1.29 (0.34)
4	3.56 (1.40)	−0.52 (0.17)	−1.02 (0.34)
5	3.17 (1.38)	−0.19 (0.17)	−1.18 (0.34)
6	2.89 (1.47)	0.12 (0.17)	−1.31 (0.34)
7	2.54 (1.38)	0.44 (0.17)	−1.04 (0.34)
8	3.86 (1.13)	−0.75 (0.17)	−0.14 (0.34)
9	2.89 (1.24)	0.03 (0.17)	−0.90 (0.34)
10	3.07 (1.27)	−0.04 (0.17)	−0.83 (0.34)
11	3.02 (1.23)	−0.09 (0.17)	−0.69 (0.34)
12	2.81 (1.07)	0.01 (0.17)	−0.51 (0.34)
13	2.68 (1.20)	0.13 (0.17)	−0.76 (0.34)
14	3.66 (1.47)	−0.72 (0.17)	0.81 (0.34)
15	3.81 (1.32)	−0.91 (0.17)	−0.45 (0.34)
16	3.87 (1.23)	−0.89 (0.17)	−0.28 (0.34)
17	3.61 (1.40)	−0.65 (0.17)	−0.90 (0.34)
18	3.76 (1.34)	−0.78 (0.17)	−0.51 (0.34)

Note: Study 3.

## Data Availability

The data supporting the conclusions of this article will be made available from the corresponding author upon reasonable request. The data are not publicly available due to ethical restrictions.

## References

[1] Cash T.F., Smolak L., Cash T.F., Smolak L. (2011). Understanding Body Images: Historical and Contemporary Perspectives. Body Image: A Handbook of Science, Practice, and Prevention.

[2] Moss T.P., Lawson V., White P. (2014). Appearance Research Collaboration. Salience and valence of appearance in a population with a visible difference of appearance: Direct and moderated relationships with self-consciousness, anxiety and depression. PLoS ONE.

[3] Nerini A., Matera C., Romani F., Di Gesto C., Policardo G.R. (2024). Retouched or unaltered? That is the question. Body image and acceptance of cosmetic surgery in Young female Instagram users. Aesthetic Plast. Surg..

[4] Thompson J.K., Coovert M.D., Stormer S.M. (1999). Body image, social comparison, and eating disturbance: A covariance structure modeling investigation. Int. J. Eat. Disord..

[5] Tylka T.L., Daniels A., Gillen M.M., Markey C.H. (2018). Overview of the field of positive body image. Body Positive: Understanding and Improving Body Image in Science and Practice.

[6] Di Gesto C., Preston C., Nerini A., Matera C., Grano C. (2025). Testing the Tripartite Influence Model on Body Image Among Pregnant Women. Body Image.

[7] Skouteris H., Bailey C., Nagle C., Hauck Y., Bruce L., Morris H. (2017). Interventions designed to promote exclusive breastfeeding in high-income countries: A systematic review update. Breastfeed. Med..

[8] Grajek M., Krupa-Kotara K., Grot M., Kujawińska M., Helisz P., Gwioździk W., Białek-Dratwa A., Staśkiewicz W., Kobza J. (2022). Perception of the Body Image in Women after Childbirth and the Specific Determinants of Their Eating Behavior: Cross-Sectional Study. Int. J. Environ. Res. Public Health.

[9] Hodgkinson E.L., Smith D.M., Wittkowski A. (2014). Women’s experiences of their pregnancy and postpartum body image: A systematic review and meta-synthesis. BMC Pregnancy Childbirth.

[10] Tavakoli M., Hasanpoor-Azghady S.B., Farahani L.A. (2021). Predictors of mothers’ postpartum body dissatisfaction based on demographic and fertility factors. BMC Pregnancy Childbirth.

[11] Clark A., Skouteris H., Wertheim E.H., Paxton S.J., Milgrom J. (2009). The relationship between depression and body dissatisfaction across pregnancy and the postpartum: A prospective study. J. Health Psychol..

[12] Lovering M.E., Rodgers R.F., George J.E., Franko D.L. (2018). Exploring the Tripartite Influence Model of body dissatisfaction in postpartum women. Body Image.

[13] Tavares I.M., Nobre P.J., Heiman J.R., Rosen N.O. (2023). Longitudinal associations between mindfulness and changes to body image in first-time parent couples. Body Image.

[14] Rodgers R.F., Hewett R.C., Nowicki G.P. (2024). A sociocultural model of the relationships between social media use and body image in midlife women. Eat. Behav..

[15] Di Gesto C., Bocci Benucci S., Policardo G.R., Maheux A.J. (2025). The Appearance-Related Social Media Consciousness Scale-Italian version (ASMCS-I) in young adults and adults. Body Image.

[16] Roth H., Homer C., Fenwick J. (2012). “Bouncing back”: How Australia’s leading women’s magazines portray the postpartum ‘body’. Women Birth J. Aust. Coll. Midwives.

[17] Rauff E.L., Downs D.S. (2011). Mediating effects of body image satisfaction on exercise behavior, depressive symptoms, and gestational weight gain in pregnancy. Ann. Behav. Med. A Publ. Soc. Behav. Med..

[18] Shloim N., Hetherington M.M., Rudolf M., Feltbower R.G. (2015). Relationship between body mass index and women’s body image, self-esteem and eating behaviours in pregnancy: A cross-cultural study. J. Health Psychol..

[19] Fern V.A., Buckley E., Grogan S. (2014). Women’s experiences of body image and baby feeding choices: Dealing with the pressure to be slender. Br. J. Midwifery.

[20] Silveira M.L., Ertel K.A., Dole N., Chasan-Taber L. (2015). The role of body image in prenatal and postpartum depression: A critical review of the literature. Arch. Women’s Ment. Health.

[21] He J., Chen X., Luo B. (2025). The association between body image and depressive symptoms in pregnant and postpartum women: A meta-analysis. Front. Public Health.

[22] Neiterman E., Fox B. (2017). Controlling the unruly maternal body: Losing and gaining control over the body during pregnancy and the postpartum period. Soc. Sci. Med..

[23] Chan C.Y., Lee A.M., Koh Y.W., Lam S.K., Lee C.P., Leung K.Y., Tang C.S.K. (2020). Associations of body dissatisfaction with anxiety and depression in the pregnancy and postpartum periods: A longitudinal study. J. Affect. Disord..

[24] Hartley E., Hill B., McPhie S., Skouteris H. (2018). The associations between depressive and anxiety symptoms, body image, and weight in the first year postpartum: A rapid systematic review. J. Reprod. Infant Psychol..

[25] Yager Z., Prichard I., Hart L., Damiano S.R. (2022). Mumbod? A comparison of body image and dietary restraint among women with younger, older, and no children. J. Health Psychol..

[26] Newby R.M., Davies P.S. (2016). Why do women stop breast-feeding? Results from a contemporary prospective study in a cohort of Australian women. Eur. J. Clin. Nutr..

[27] Gjerdingen D., Fontaine P., Crow S., McGovern P., Center B., Miner M. (2009). Predictors of mothers’ postpartum body dissatisfaction. Women Health.

[28] Morley-Hewitt A.G., Owen A.L. (2020). A systematic review examining the association between female body image and the intention, initiation and duration of post-partum infant feeding methods (breastfeeding vs. bottle-feeding). J. Health Psychol..

[29] Brown A., Rance J., Warren L. (2015). Body image concerns during pregnancy are associated with a shorter breast feeding duration. Midwifery.

[30] Harder T., Bergmann R., Kallischnigg G., Plagemann A. (2005). Duration of breastfeeding and risk of overweight: A meta-analysis. Am. J. Epidemiol..

[31] Rossiter M.D., Colapinto C.K., Khan M.K., McIsaac J.L., Williams P.L., Kirk S.F., Veugelers P.J. (2015). Breast, Formula and Combination Feeding in Relation to Childhood Obesity in Nova Scotia, Canada. Matern. Child Health J..

[32] Quigley M.A., Kelly Y.J., Sacker A. (2007). Breastfeeding and hospitalization for diarrheal and respiratory infection in the United Kingdom Millennium Cohort Study. Pediatrics.

[33] Li D.P., Du C., Zhang Z.M., Li G.X., Yu Z.F., Wang X., Li P.F., Cheng C., Liu Y.P., Zhao Y.S. (2014). Breastfeeding and ovarian cancer risk: A systematic review and meta-analysis of 40 epidemiological studies. Asian Pac. J. Cancer Prev..

[34] Aune D., Norat T., Romundstad P., Vatten L.J. (2014). Breastfeeding and the maternal risk of type 2 diabetes: A systematic review and dose-response meta-analysis of cohort studies. Nutr. Metab. Cardiovasc. Dis. NMCD.

[35] Peters S.A.E., Yang L., Guo Y., Chen Y., Bian Z., Du J., Yang J., Li S., Li L., Woodward M. (2017). Breastfeeding and the Risk of Maternal Cardiovascular Disease: A Prospective Study of 300 000 Chinese Women. J. Am. Heart Assoc..

[36] Alleva J.M., Tylka T.L. (2021). Body functionality: A review of the literature. Body Image.

[37] Matera C., Casati C., Paradisi M., Di Gesto C., Nerini A. (2024). Positive Body Image and Psychological Wellbeing Among Women and Men: The Mediating Role of Body Image Coping Strategies. Behav. Sci..

[38] Swami V., Barron D., Hari R., Grover S., Smith L., Furnham A. (2019). The nature of positive body image: Examining associations between nature exposure, self-compassion, functionality appreciation, and body appreciation. Ecopsychology.

[39] Linardon J., McClure Z., Tylka T.L., Fuller-Tyszkiewicz M. (2022). Body appreciation and its psychological correlates: A systematic review and meta-analysis. Body Image.

[40] Liu C., Jarman H.K., Messer M., Linardon J. (2025). Predictors of functionality appreciation: Prospective findings. Body Image.

[41] Rubin L.R., Steinberg J.R. (2011). Self-objectification and pregnancy: Are body functionality dimensions protective?. Sex Roles A J. Res..

[42] Rosenbaum D.L., Gillen M.M., Markey C.H. (2020). Feeling let down: An investigation of breastfeeding expectations, appreciation of body functionality, self-compassion, and depression symptoms. Appetite.

[43] Di Gesto C., Matera C., Nerini A., Policardo G.R., Stefanile C. (2020). Misurare le attività relative alle immagini su Instagram e il confronto relativo all’apparenza: Validazione della Instagram Image Activity Scale e della Instagram Appearance Comparison Scale. Psicol. Della Salut..

[44] Nerini A., Di Gesto C., Lo Bartolo M., Innocenti A., Stefanile C., Matera C. (2024). Self-awareness and social influences as predictors of body dissatisfaction and acceptance of cosmetic surgery for social reasons among men. Aesthetic Plast. Surg..

[45] Cash T.F., Phillips K.A., Santos M.T., Hrabosky J.I. (2004). Body Image Disturbance Questionnaire (BIDQ).

[46] Cooper P.J., Taylor M.J., Cooper Z., Fairburn C.G. (1987). Body Shape Questionnaire (BSQ).

[47] Franzoi S.L., Shields S.A. (1984). The Body Esteem Scale: Multidimensional structure and sex differences in a college population. J. Personal. Assess..

[48] Alleva J.M., Tylka T.L., Kroon Van Diest A.M. (2017). The Functionality Appreciation Scale (FAS): Development and psychometric evaluation in U.S. community women and men. Body Image.

[49] Peterson C. (2000). The future of optimism. Am. Psychol..

[50] Williams E.F., Cash T.F., Santos M.T. (2004). Positive and negative body image: Precursors, correlates, and consequences. 38th Annu. Assoc. Adv. Behav. Ther..

[51] Avalos L.C., Tylka T.L., Wood-Barcalow N. (2005). The Body Appreciation Scale: Development and psychometric evaluation. Body Image.

[52] Dimitrov D.M. (2012). Statistical Methods for Validation of Assessment Scale Data in Counseling and Related Fields. Appl. Psychol. Meas..

[53] Kyriazos T., Stalikas A. (2018). Applied Psychometrics: The Steps of Scale Development and Standardization Process. Psychology.

[54] Bland J.M., Altman D.G. (1986). Statistical methods for assessing agreement between two methods of clinical measurement. Lancet.

[55] Cicchetti D.V., Sparrow S.A. (1981). Developing criteria for establishing interrater reliability of specific items: Applications to assessment of adaptive behavior. Am. J. Ment. Defic..

[56] (2019). The Jamovi Project. https://www.jamovi.org.

[57] Swami V., Barron D. (2019). Translation and validation of body image instruments: Challenges, good practice guidelines, and reporting recommendations for test adaptation. Body Image.

[58] Boateng G.O., Neilands T.B., Frongillo E.A., Melgar-Quiñonez H.R., Young S.L. (2018). Best Practices for Developing and Validating Scales for Health, Social, and Behavioral Research: A Primer. Front. Public Health.

[59] Coyne I., Holmström I., Söderbäck M. (2018). Centeredness in Healthcare: A Concept Synthesis of Family-centered Care, Person-centered Care and Child-centered Care. J. Pediatr. Nurs..

[60] Gillen M.M., Markey C.H., Rosenbaum D.L., Dunaev J.L. (2021). Breastfeeding, body image, and weight control behavior among postpartum women. Body Image.

[61] Riesco-González F.J., Antúnez-Calvente I., Vázquez-Lara J.M., Rodríguez-Díaz L., Palomo-Gómez R., Gómez-Salgado J., García-Iglesias J.J., Parrón-Carreño T., Fernández-Carrasco F.J. (2022). Body image dissatisfaction as a risk factor for postpartum depression. Medicina.

[62] (2020). Qualtrics. Provo, UT, USA. https://www.qualtrics.com.

[63] Nunnally J. (1978). Psychometric Theory.

[64] Kaiser H. (1974). An Index of Factorial Simplicity. Psychometrika.

[65] Bartlett M.S. (1950). Tests of significance in factor analysis. Br. J. Psychol..

[66] Fabrigar L.R., Wegener D.T., MacCallum R.C., Strahan E.J. (1999). Evaluating the use of exploratory factor analysis in psychological research. Psychol. Methods.

[67] Goretzko D., Bühner M. (2020). One model to rule them all? Using machine learning algorithms to determine the number of factors in exploratory factor analysis. Psychol. Methods.

[68] Costello A.B., Osborne J. (2019). Best practices in exploratory factor analysis: Four recommendations for getting the most from your analysis. Pract. Assess. Res. Eval..

[69] Kaiser H.F. (1960). The application of electronic computers to factor analysis. Educ. Psychol. Meas..

[70] Curran P.J., West S.G., Finch J.F. (1996). The Robustness of Test Statistics to Non-Normality and Specification Error in Confirmatory Factor Analysis. Psychol. Methods.

[71] Clark L.A., Watson D. (2019). Constructing validity: New developments in creating objective measuring instruments. Psychol. Assess..

[72] McKinley N.M., Hyde J.S. (1996). The objectified body consciousness scale: Development and validation. Psychol. Women Q..

[73] Dowson J., Henderson L. (2001). The validity of a short version of the Body Shape Questionnaire. Psychiatry Res..

[74] Erdfelder E., Faul FBuchner A. (1996). GPOWER: A general power analysis program. Behav. Res. Methods Instrum. Comput..

[75] Dakanalis A., Carrà G., Calogero R., Fida R., Clerici M., Zanetti M.A., Riva G. (2015). The developmental effects of media-ideal internalization and self-objectification processes on adolescents’ negative body-feelings, dietary restraint, and binge eating. Eur. Child Adolesc. Psychiatry.

[76] Cerea S., Todd J., Ghisi M., Mancin P., Swami V. (2021). Psychometric properties of an Italian translation of the Functionality Appreciation Scale (FAS). Body Image.

[77] Matera C., Nerini A., Stefanile C. (2013). Assessing body dissatisfaction: Validation of the Italian version of the Body Shape Questionnaire-14 (BSQ-14)]. Couns. G. Ital. Di Ric. E Appl..

[78] Grano C., Fernandes M., Conner M. (2023). Predicting intention and maintenance of breastfeeding up to 2-years after birth in primiparous and multiparous women. Psychol. Health.

[79] West S.G., Finch J.F., Curran P.J., Hoyle R.H. (1995). Structural equation models with non-normal variables: Problems and remedies. Structural Equation Modeling: Concepts, Issues, and Applications.

[80] Browne M.W., Cudeck R. (1992). Alternative Ways of Assessing Model Fit. Sociol. Methods Res..

[81] Wheaton B., Muthen B., Alwin D.F., Summers G. (1977). Assessing Reliability and Stability in Panel Models. Sociol. Methodol..

[82] Bergstrom N., Braden B., Kemp M., Champagne M., Ruby E. (1998). Predicting pressure ulcer risk: A multisite study of the predictive validity of the Braden Scale. Nurs. Res..

[83] Cohen J. (1988). Statistical Power Analysis for the Behavioral Sciences.

[84] DiStefano C., Motl R.W. (2006). Further investigating method effects associated with negatively worded items on self-report surveys. Struct. Equ. Model..

[85] Horan P.M., DiStefano C., Motl R.W. (2003). Wording effects in self-esteem scales: Methodological artifact or response style?. Struct. Equ. Model..

[86] Byrne B.M. (2004). Testing for multigroup invariance using AMOS graphics: A road less traveled. Struct. Equ. Model..

[87] O’Malley D., Higgins ASmith V. (2021). Exploring the Complexities of Postpartum Sexual Health. Curr. Sex. Health Rep..

[88] Watson B., Fuller-Tyszkiewicz M., Broadbent J., Skouteris H. (2015). The meaning of body image experiences during the perinatal period: A systematic review of the qualitative literature. Body Image.

[89] Hartley E., Fuller-Tyszkiewicz M., Skouteris H., Hill B. (2021). A qualitative insight into the relationship between postpartum depression and body image. J. Reprod. Infant Psychol..

[90] Hopper K.M., Aubrey J.S. (2016). Bodies After Babies: The Impact of Depictions of Recently Post-Partum Celebrities on Non-Pregnant Women’s Body Image. Sex Roles.

[91] Singh Solorzano C., Porciello G., Violani C., Grano C. (2022). Body image dissatisfaction and interoceptive sensibility significantly predict postpartum depressive symptoms. J. Affect. Disord..

[92] Fredrickson B.L., Roberts T.A. (1997). Objectification theory: Toward understanding women’s lived experiences and mental health risks. Psychol. Women Q..

[93] Ferreira C., Dias B., Oliveira S. (2019). Behind women’s body image-focused shame: Exploring the role of fears of compassion and self-criticism. Eat. Behav..

[94] Jackson T., Chen H. (2015). Features of objectified body consciousness and sociocultural perspectives as risk factors for disordered eating among late-adolescent women and men. J. Couns. Psychol..

[95] Mills J.S., Minister C., Samson L. (2022). Enriching sociocultural perspectives on the effects of idealized body norms: Integrating shame, positive body image, and self-compassion. Front. Psychol..

[96] Rodgers R.F., McLean S.A., Marques M., Dunstan C.J., Paxton S.J. (2016). Trajectories of Body Dissatisfaction and Dietary Restriction in Early Adolescent Girls: A Latent Class Growth Analysis. J. Youth Adolesc..

[97] Boybay Koyuncu S., Duman M. (2022). Body dissatisfaction of women during postpartum period and coping strategies. Women Health.

